# *ABrox*—A user-friendly Python module for approximate Bayesian computation with a focus on model comparison

**DOI:** 10.1371/journal.pone.0193981

**Published:** 2018-03-08

**Authors:** Ulf Kai Mertens, Andreas Voss, Stefan Radev

**Affiliations:** Department of Psychology, Heidelberg University, Heidelberg, Germany; Southwest University, CHINA

## Abstract

We give an overview of the basic principles of approximate Bayesian computation (ABC), a class of stochastic methods that enable flexible and likelihood-free model comparison and parameter estimation. Our new open-source software called *ABrox* is used to illustrate ABC for model comparison on two prominent statistical tests, the two-sample t-test and the Levene-Test. We further highlight the flexibility of ABC compared to classical Bayesian hypothesis testing by computing an approximate Bayes factor for two multinomial processing tree models. Last but not least, throughout the paper, we introduce *ABrox* using the accompanied graphical user interface.

## Introduction

Approximate Bayesian computation (ABC) is a computational method founded in Bayesian statistics. ABC enables parameter estimation as well as model comparison when the probability of observed data under the model of consideration is unknown, or put differently, when the likelihood function is not available or computationally intractable. The idea of ABC is to bypass the computation of the likelihood by comparing model predictions with observed data, thus taking a simulation-based approach.

In psychological research, the classical null hypothesis significance testing (NHST) based on the *p*-value falls more and more under disrepute due to its many weaknesses [1–6]. One main criticism among others, especially stressed by Wagenmakers and colleagues [7], is the fact that *p*-values only depend on what is expected under the null hypothesis ($H_{0}$) but not what is expected under the alternative hypothesis ($H_{1}$). Hence, a small *p*-value only states that the observed data is unlikely under $H_{0}$ but it might be even more unlikely under $H_{1}$. Furthermore, the *p*-value is often misinterpreted (e.g. as the probability of the null hypthesis being true) [8]. As a solution, many authors suggest the use of Bayes factors as they allow to quantify evidence for both $H_{1}$ and $H_{0}$ and can be easily interpreted as the relative evidence of one hypothesis with respect to another hypothesis [1, 2, 4, 7, 8].

Despite the advantages of Bayes factors, a shortcoming is that their computation is often hard or even impossible which in turn narrows their applicability [9]. Wagenmakers and colleagues [5] address this aspect by stating:

“In order to profit from the practical advantages that Bayesian parameter estimation and Bayes factor hypothesis tests have to offer it is vital that the procedures of interest can be executed in accessible, user-friendly software package”

(p. 4).

They provide such a software package, called JASP [10], in order to ease the process of Bayesian hypothesis-testing. With JASP, they continue,

“[…] users are able to conduct classical analyses as well as Bayesian analyses, without having to engage in computer programming or mathematical derivation”

(p. 4).

Yet, users of JASP are restricted when it comes to the specification of user-defined prior distributions or the application of non-standard tests. In addition, users are limited in the application of Bayesian hypothesis-testing in the sense that JASP always assumes a point null hypothesis and thus the models being compared are always nested. Although such a scenario is undoubtedly the most common one in psychological research, it is desirable to extend the methods for Bayesian model comparison to allow for non-nested model comparison. *ABrox* exactly addresses these issues by providing a flexible toolbox to estimate Bayes factors in areas where an exact mathematical solution is not available.

The outline of the paper is as follows. First, we will give an introduction to ABC with a focus on model comparison (technical details on ABC can be found elsewhere, [11, 12] or [13]). We will then introduce our software called *ABrox* and demonstrate its validity by analysing two models. For this purpose, we choose the Bayesian two-sample t-test and the Levene test. Although there exists a bayesian version of the Levene test [14], a default Bayes factor is only available for the two-sample t-test [15]. Lastly, we show the flexibility of *ABrox* by comparing two multinomial processing tree models applied to the weapon-misidentification task [16, 17].

## Approximate Bayesian computation for model selection

As previously mentioned, ABC can be used for both parameter estimation and model comparison. If one is interested in the latter, the general idea of ABC methods is to test how well simulated data from different models can mimic the observed data. If a model does not represent the true data-generating process of the observed data reasonably well, then simulated data from this model will not resemble the observed data adequately. Imagine for instance that some data follow a quadratic trend. Trying to simulate such data with a model assuming a linear trend will fail most of the time. After multiple runs of the algorithm, the models are compared by counting the number of times each one was able generate data that resemble the observed data sufficiently well. One common algorithm is the ABC rejection algorithm for model selection, in its basic form introduced by Rubin (1984) [18] (see explanation below):

1. Draw m* from the prior *P(m)*
2. Sample θ* from the prior *P*(*θ* | m*)
3. Simulate a candidate dataset D* ∼ *f*(*D* | θ*, m*)
4. Compute the distance. If *d*(*D*_0_, D*) ≤ *ϵ*, accept (m*, θ*), otherwise reject it.
5. Return to 1 until *k* particles are accepted.

First (step 1), one of the models under consideration (m*) is picked based on the (prior-)probabilities of the models. In step 2, given the model, parameters (θ*) from the prior distributions of the parameters from the chosen model *m* (*P*(*θ* | m*)) are drawn. Using model and parameters, data (D*) is simulated in step 3 and is then compared with the observed data in step 4. For this purpose, a distance measure *d* has to be defined that quantifies the dissimilarity of simulated and observed data. If the distance *d* exceeds a predefined threshold (*ϵ*), the so-called particle containing both model indicator and the sampled parameters (m*, θ*) is accepted and stored. The process is repeated *N* times until *k* particles have been accepted. The number of accepted particles is then divided by *N* to get a marginal posterior distribution of a model (m’) stating how likely the model is given the data at hand (*D*_0_) [11].

$$P\left(m^{\prime }\;|\;D_{0}\right)\approx \frac{\#\;accepted\;particles(m^{\prime },.)}{N}$$ (1)

With this metric, we can calculate the Bayes factor (BF) as the ratio of marginal likelihoods (*p*(*D*_0_ | *m*_1_)/*p*(*D*_0_ | *m*_2_)). The Bayes factor (*BF*_12_) describes the amount of change from prior odds to posterior odds after the data has been observed [5].

$$\begin{array}{c} \frac{p(D_{0}\;|\;m_{1})}{p(D_{0}\;|\;m_{2})}=\frac{p(m_{1}\;|\;D_{0})}{p(m_{2}\;|\;D_{0})}\times \frac{p(m_{2})}{p(m_{1})} \end{array}$$ (2)

In Eq 2, *p*(*m*_2_)/*p*(*m*_1_) is referred to the model prior odds, indicating how likely one model (*m*_2_) is relative to the other (*m*_1_) before seeing the data. *p*(*m*_1_ | *D*_0_)/*p*(*m*_2_ | *D*_0_) is the ratio of marginal posterior distributions as approximated via ABC.

### Summary statistics

In step 4 of the aforementioned algorithm, the distance could be computed by taking every data point in the observed and simulated dataset into account. While computing the distance based on every data point is reasonable theoretically, it is infeasible in most settings. If the datasets are large, then there is always a deviation from simulated data to observed data (the distance would only be zero if every simulated data point was exactly equal to the corresponding observed one). Hence, the acceptance rates would be extremely low if the distance threshold (*ϵ*) was too strict. However, simply increasing the distance threshold to account for the low acceptance rates would not be a reasonable decision either because exceedingly large thresholds distort the approximation [19]. In order to account for these problems, the distance *d* between observed and simulated data is often based on a summary statistic *s*(*D*) (e.g., mean and (co)variance). While this procedure of computing summary statistics suffers less from the *curse of dimensionality*, it often comes with a loss of information. Didelot et al. [12] showed that insufficient summary statistics, not capturing the whole information of the raw data, can lead to inconsistencies. Hence, the ABC model selection may fail to recover the true model. The challenge is to balance out consistency and information loss [12]. Therefore, the approximation of the true Bayes factor depends on the summary statistic capturing the necessary information in the data.

### The threshold *ϵ*

Besides picking an appropriate summary statistic, the notion of simulated data being “close enough” to observed data has to be defined. Users interested in ABC have to set a specific threshold value *ϵ* based on a distance metric in order to decide whether a particle (m*, θ*) should be accepted or discarded. The smaller the threshold *ϵ*, the better the approximation of the marginal posterior distribution to the true posterior distribution [11]. If the threshold is too liberal, too many particles fall below the threshold and the resulting posterior distribution is not a good approximation. If, on the other hand, the threshold is too strict, it might take very long to find the required number of particles that lead to distances smaller than *ϵ* and the procedure becomes computationally inefficient. The challenge is to find an *ϵ* that leads to a good approximation while retaining a tolerable running time of the algorithm. To tackle the problem of finding a good threshold, Beaumont, Cornuet, Marin, and Robert [20] proposed to select *ϵ* as the *k*^*th*^ percentile of computed distances prior to the actual start of the algorithm (with *k* being a small value such as 5 or 1).

## Introducing *ABrox*

We developed *ABrox* as an open-source python module which enables approximate Bayesian model comparison and parameter estimation. With *ABrox*, we introduce a graphical user interface (GUI) which is designed to be used as a tool for all-purpose ABC, making the methods much more accessible to researchers interested in applying ABC. The GUI is especially useful for researchers without much prior knowledge of ABC. All necessary steps are separated into small chunks to keep the structure clear and simple. Users can also save and load their projects which allows for good maintenance and easy sharing with collaborators. Moreover, prior distributions are automatically visualized for a better user-experience. *ABrox* facilitates high flexibility regarding the specification of mathematical models and is especially useful if one is interested in Bayesian model comparison where none of the traditional methods are suitable, usual assumptions underlying those methods are not met, models of interest are not nested, or software does not permit to change prior settings. Detailed instructions on how to install *ABrox* can be found online (see https://github.com/mertensu/ABrox).

### Comparison to other software packages

The main advantages of *ABrox* compared to other software packages such as the R-package abc [21] or the Python module ABC-SysBio [22] are the domain-independence, meaning that it is not designed for a specific field of research, and its ease of use due to the GUI. The software *DIYABC* [23] is another attempt at simplifying the use of ABC by providing a GUI for experimental biologists. *DIYABC* provides a convenient way to conduct ABC inferences about population history using SNPs (Single Nucleotide Polymorphism), DNA sequence and microsatellite data, the areas of research it was designed for. *DIYABC* does not, as *ABrox*, necessitate prior experience with a programming language. Due to its focus on specialized fields of research, the process of simulating data and computing summary statistics is handled by the program itself which is a helpful feature for users. However, it is limited to a few specific fields of research.

### The ABC reference table

The *reference table* in ABC is the starting point for most algorithms. It is a large table containing in each row the the model index (only used for model comparison), summary statistics of a simulated dataset, the parameter(s) used for simulating the data and the distance with respect to the observed summary statistics. The *reference table* contains as many rows as there are simulated datasets. *ABrox* usually generates the *reference table* internally but it also possible to import an external *reference table* stored in a *comma separated value* (csv) file. The header of csv-file needs to satisfy the the following conditions. There has to be a column named *idx* containing the model index. Furthermore, if there are *k* summary statistics, each value has to be stored in separate columns named *s_* followed by a number (usually 1 to *k*). The same logic applies to the parameters where each parameter column name should start with *p_*. In addition to the columns containing information about summary statistics and parameter prior distributions, there has to be a column named *distance* containing the distance to the observed summary statistics.

### Features of *ABrox*

#### Implemented algorithms

For model comparison, either the rejection algorithm or a random forest approach can be chosen. Random forests [24] are a supervised learning algorithm used extensively in machine learning. Given a sample of input-output pairs, the goal of a random forest is to learn a mapping from the input to the output by training multiple decision trees (a standard non-parametric algorithm for classification and regression) and aggregating their decision function outputs. It incorporates the techniques bootstrap aggregation and random feature selection to reduce over-fitting and thus increases the predictive generalization of the model. In the context of ABC, random forests learns to map summary statistics to either model indices or parameters of a model. Readers interested in more details should consult the work by Pudlo et al. (2015) [25]. Besides model comparison, parameter estimation can be conducted either via the basic rejection algorithm or a Markov chain Monte Carlo (MCMC) based algorithm by Wegmann [26].

#### Cross-validation

*ABrox* allows for cross-validating the results from both model comparison and parameter estimation using leave-one-out cross-validation as used in [21]. In leave-one-out cross-validation, a randomly chosen simulated summary statistic is treated as pseudo-observed summary statistic and an ABC algorithm is run in order to estimate parameters or a Bayes factor (posterior model probability). This procedure is repeated *N* times. In the case of parameter estimation, *ABrox* then provides a prediction error (see Eq 3) stating how different the predicted parameters ($\tilde{\theta }$) are from the true parameters (*θ*).

$$\begin{array}{c} E_{pred}=\frac{\sum_{i}{\left(\tilde{\theta_{i}}-\theta_{i}\right)}^{2}}{Var(\theta_{i})} \end{array}$$ (3)

Because the information provided by Eq 3 is limited, an additional pdf-file is automatically saved. This file contains one plot for each parameter in which the estimated parameter is plotted against the true parameter.

When performing model comparison, *ABrox* presents a confusion matrix stating how often the pseudo-observed summary statistics could be correctly classified to the model they were generated from. In this matrix, the diagonal elements should be large and the values in off-diagonal elements should be small. The confusion matrix is also stored in a heatmap-style as a pdf-file to ease the interpretation of the results.

### The building blocks of *ABrox*

To calculate a Bayes factor, the user needs to provide several pieces of information.

1. the data to be analyzed (optional)
2. the prior distributions for all parameters of all models.
3. functions to simulate data from the models.
4. a function to compute summary statistics from data.
5. a function to compute the distance between summary statistics of observed and simulated data (optional).

The functions to compute the summary-statistic and the distance are the same for all compared models. Prior distributions are internally stored as python dictionaries whereas simulation-, summary-, and distance-function are user-defined functions.

#### Data import in *ABrox*

The data tab in *ABrox* shown in S1 Fig is used to import an external data-file. The file should be stored as a comma-separated value file (*.csv*). The imported data can be inspected and modified in the data tab. Note that users are able to access the data in the embedded Python Console at the bottom by typing *data*.

#### The prior distribution in *ABrox*

Prior distributions for the parameters of each model have to be specified. S2 Fig shows the prior settings tab of *ABrox* with a parameter called *x* and its corresponding prior distribution showing a standard normal distribution. In the current version of *ABrox*, a total of nine different prior distributions are available.

#### The simulate-function in *ABrox*

In a next step, the user has to write a Python function for each model that simulates data. The simulated dataset has to be in the form of a NumPy array [27]. The simulate-function can be written in the *simulate* tab (S3 Fig). Note that the names of the functions should not be changed and default to *simulate*. The function has only one argument (*params*) which is a dictionary with keys corresponding to the names of the parameters specified and the values containing one sampled value from the respective prior distribution of the parameter. Thus, each parameter value can easily be accessed by the parameter’s name.

The user has to take care that the format of the imported (observed) data is identical to the format of the simulated data. Put differently, the return type of the simulate-function has to be the same as the type of the imported data. If, for instance, the observed data is a multidimensional array with 100 rows and two columns, the simulate function has to return a multidimensional array of the same shape. If this is not the case, *ABrox* will throw an error message.

#### The summary and distance-function in *ABrox*

After the specification of each model (priors and *simulate*-function), a new Python function to calculate the summary statistics from the data has to be written. The function takes as input the output of the simulate function specified in the previous step. The function should return the summary statistics. The summary statistics can be a scalar or a vector.

In *ABrox*, the user can optionally write a user-defined distance function for computing the distance between observed summary statistics and simulated summary statistics. If the function is left empty, *ABrox* automatically uses the default distance metric which is the Euclidean distance scaled on the median absolute deviation (MAD).

#### The settings tab in *ABrox*

In the settings tab (S4 Fig), the working directory has to be set. Next, the type of analysis, that is model comparison or parameter estimation, has to be selected. Note that *ABrox* allows to compare more than two models by simply adding more models to the *Project Tree*. Each model needs to be specified with a unique *simulate*-function and the corresponding prior distribution(s) for the parameter(s). If the rejection algorithm is selected, *ABrox* calculates a matrix containing approximate Bayes factors. The random forest approach, on the other hand, calculates posterior model probabilities.

In the case of parameter estimation, a summary of the posterior distributions of the parameters is returned. In addition, a data frame with all samples from the posterior of each parameter is saved in the working directory.

The *Console Panel* at the bottom of S4 Fig shows the progress of the computation and informs the user as soon as the computation is finished. The results are stored inside a python variable which can be accessed from the integrated *Python Console*.

The user interface of *ABrox* generates a python script in the working directory with all the configurations specified in the GUI. This feature of saving a python-file with all the configurations is especially useful if users want to use *ABrox* within the Python framework.

Besides the obligatory settings, we added the option to generate pseudo-observed data from a model instead of having to import data (*Model Test Settings*). By choosing this option, one can simply check if the approximate Bayes factor favors the model the data have been simulated from or if the parameters chosen for the pseudo-observed data can be estimated accurately.

## Application example 1: The independent-samples t-test

In the following, we will first show how to compute an approximate Bayes factor for an independent samples t-test assuming normally distributed data with equal variances. The corresponding *ABrox* project file can be downloaded at https://github.com/mertensu/ABrox/tree/master/project_files. We chose the t-test as the first example since it is one of the most common tests in statistics. Furthermore, the t-test qualifies for a comparison of the approximate Bayes factor and the default Bayes factor as there is already software available to compute a default Bayes factor for the t-test [15]. For all of the following examples, we refer to *Bayes factor* as the Bayes factor expressing support for the alternative hypothesis ($H_{1}$) over the null hypothesis ($H_{0}$), which is usually indicated as *BF*_10_.

### Model specification

#### Simulation

In order to adapt the default Bayesian t-test for *ABrox*, we have to consider how data are simulated from both $H_{0}$ and $H_{1}$. In a first step, we know that data from both samples are drawn from a normal distribution with a specific standard deviation (*σ*) and mean (*μ*). $H_{0}$ assumes that there is no difference in means (*μ*_1_ = *μ*_2_). As a consequence, we restrict $H_{0}$ to generate two normally distributed samples with the same mean. The model representing $H_{1}$, in comparison, is allowed to simulate data such that the two means differ.

#### Summary statistic

After that, a useful summary statistic has to be chosen. In the t-test scenario, we choose the empirical effect size Cohen’s *d* as the summary statistic on which the comparison between observed data and simulated data is based [28]. Note that by comparing the data only by the effect size *d*, we do not get any information about other parameters that might be of interest in a parameter estimation scenario (such as the overall standard deviation) but focus only on model comparison.

#### Parameters and priors

**Fig 1:**
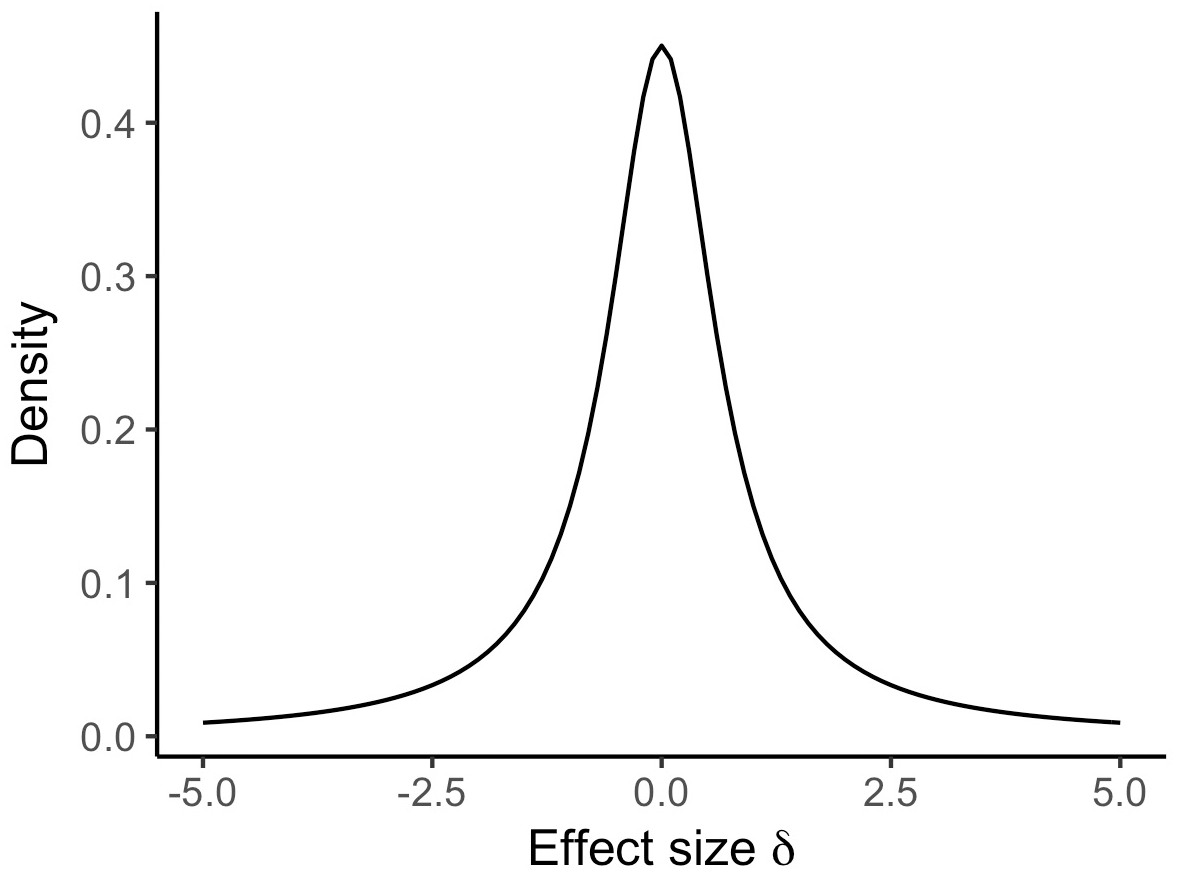
Cauchy distribution with a scale of *γ* = 0.707 used as the prior distribution.

Following Rouder et al. [15], $H_{1}$ gets a prior for the population effect size Cohen’s *d*. Whereas $H_{0}$ does not have a parameter and therefore no prior, we put a Cauchy prior with scale *γ = 0.707* on the effect size *d* for $H_{1}$ (see Fig 1).

#### Distance function

In order to assess the similarity between the observed and simulated Cohen’s *d*, the squared difference is chosen as the distance metric.

### Simulation results

In the following section, we provide simulation results focusing on the similarity between the default Bayes factor and the approximated Bayes factor. We simulated N = 1000 data sets with an Cohen’s *d* varying uniformly between no effect (*d* = 0) and a moderate effect of *d* = 0.5. For each generated data set, we computed both the default Bayes factor using the R package BayesFactor (version 0.9.12.2) [29], as well as the approximated Bayes factor using *ABrox*. Furthermore, we varied the sample size between *N*_*total*_ = 50 (25 per group) and *N*_*total*_ = 200 (100 per group).

#### Support for $H_{1}$

**Fig 2:**
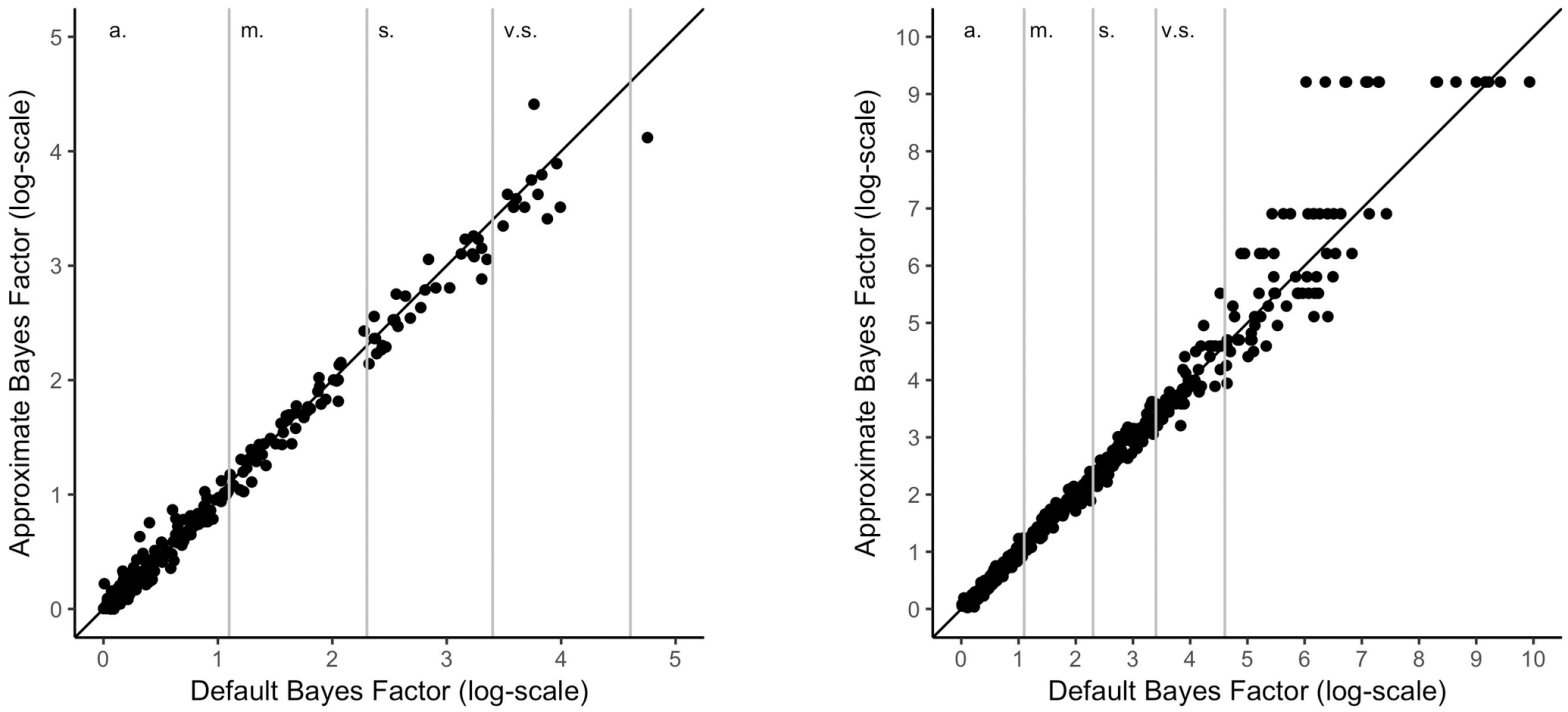
Relationship of default Bayes factor and approximated Bayes factor for a two-sample t-test with support for the alternative hypothesis; left: N = 50, right: N = 200. *Note*. a. = anecdotal. m. = moderate. s. = strong. v.s. = very strong evidence.

Results in Fig 2 indicate that, in cases where there is support for $H_{1}$, the approximation to the default Bayes factor is extremely good. There is a strong correlation when the default Bayes factor lies somewhere between anecdotal and very strong support for the $H_{1}$. In areas where there is extreme support for $H_{1}$ (to the right of very strong support in Fig 2, the accuracy of the approximation decreases somewhat). Even though this pattern of increasing differences is not optimal, it is not a serious issue since it does not matter that much how extreme the support for $H_{1}$ actually is. Note that if the null hypothesis ($H_{0}$) did not cross the threshold at all (no data could be simulated that were close enough to the observed data), this indicates extreme evidence for $H_{1}$. In this case, no ratio could be computed (division by zero). In such cases, we set the approximated Bayes factor to a value of 10000 (resulting in ln(10000) = 9.21) which is an arbitrary choice simply reflecting a very large number.

#### Support for $H_{0}$

**Fig 3:**
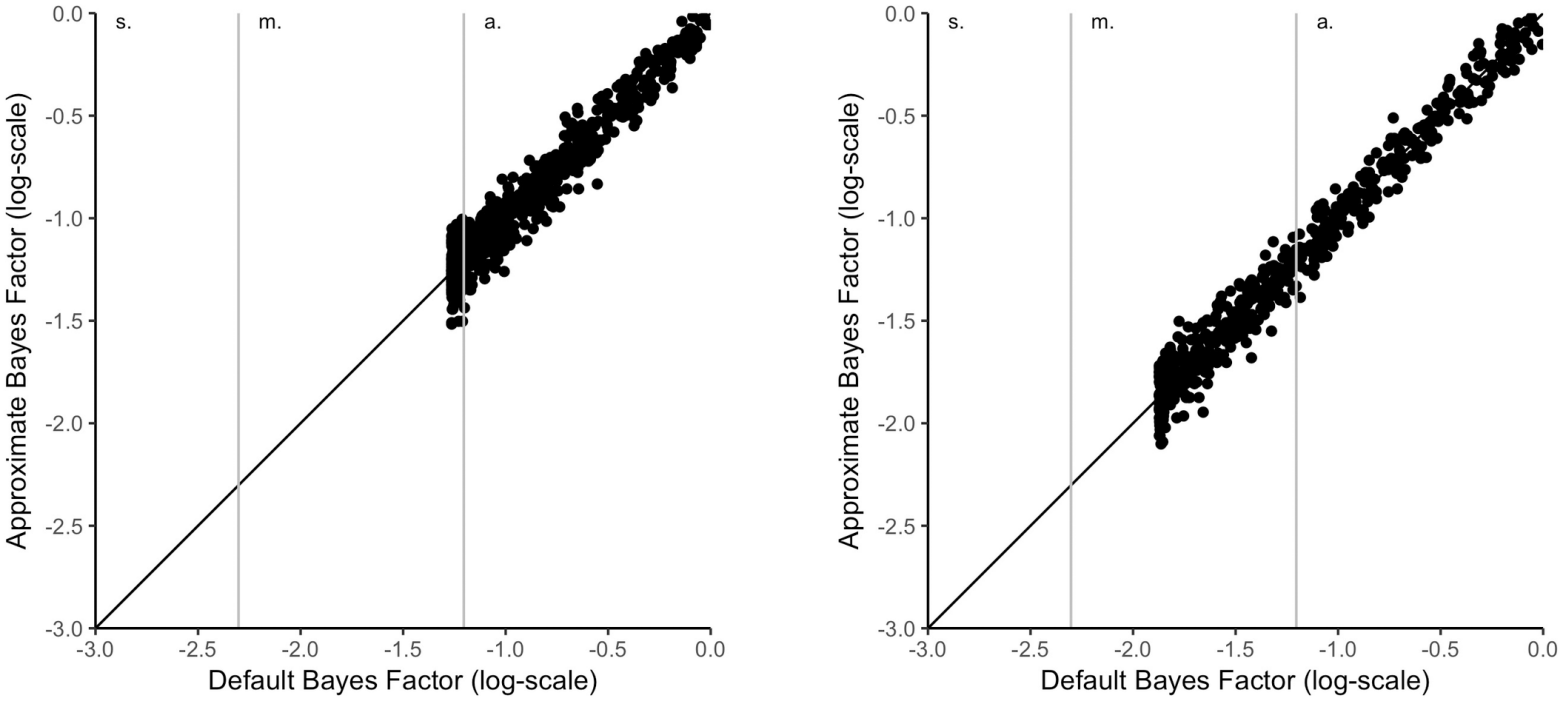
Relationship of default Bayes factor and approximated Bayes factors for a two-sample t-test with support for the null hypothesis; left: N = 50, right: N = 200. *Note*. a. = anecdotal. m. = moderate. s. = strong. v.s. = very strong evidence.

Fig 3 shows the relationship between the Bayes factors when there is support for $H_{0}$. There are two things to notice. First, there is again a very high correlation between the default and the approximated Bayes factor. Secondly, a lower limit of the default Bayes factor can be seen if the difference in means is exactly zero. One might very well ask why a mean difference of zero does not result in a Bayes factor with strong evidence for $H_{0}$. The reason for this is that the prior for $H_{1}$ also puts mass on an effect of zero so that it is still possible for the model to account for the findings. However, the more vague the prior (increasing the scale) or the larger the sample sizes, the lower the limit of the Bayes factor becomes. The lower limit of the Bayes factor in the left plot of Fig 3 is (*log*(*BF*_10_) = -1.26) and (*log*(*BF*_10_) = -1.87) for the right plot.

## Application example 2: A Bayesian Levene test

In this section, we show the flexibility of ABC model comparison by implementing a Bayesian Levene test for two samples. The corresponding *ABrox* project file can be downloaded at https://github.com/mertensu/ABrox/tree/master/project_files. The Levene test checks whether variances among different samples are equal (homogeneous). Assuming two samples, the null hypothesis ($H_{0}$) is specified as:

$$\begin{array}{c} H_{0}:\sigma_{1}^{2}=\sigma_{2}^{2} \end{array}$$ (4)

Using the ABrox framework, we construct two models. $H_{0}$ is restricted in the sense that both population variances are homogeneous. For the alternative hypothesis ($H_{1}$) however, the variances between both groups are allowed to differ. Note that the $H_{0}$ has one parameter (the identical variance within groups) whereas $H_{1}$ has two parameters (one variance for each sample). Although this parameterization is valid, we reparameterized both models such that $H_{0}$ has no parameter (we fixed the variance in both groups to 1).

**Fig 4:**
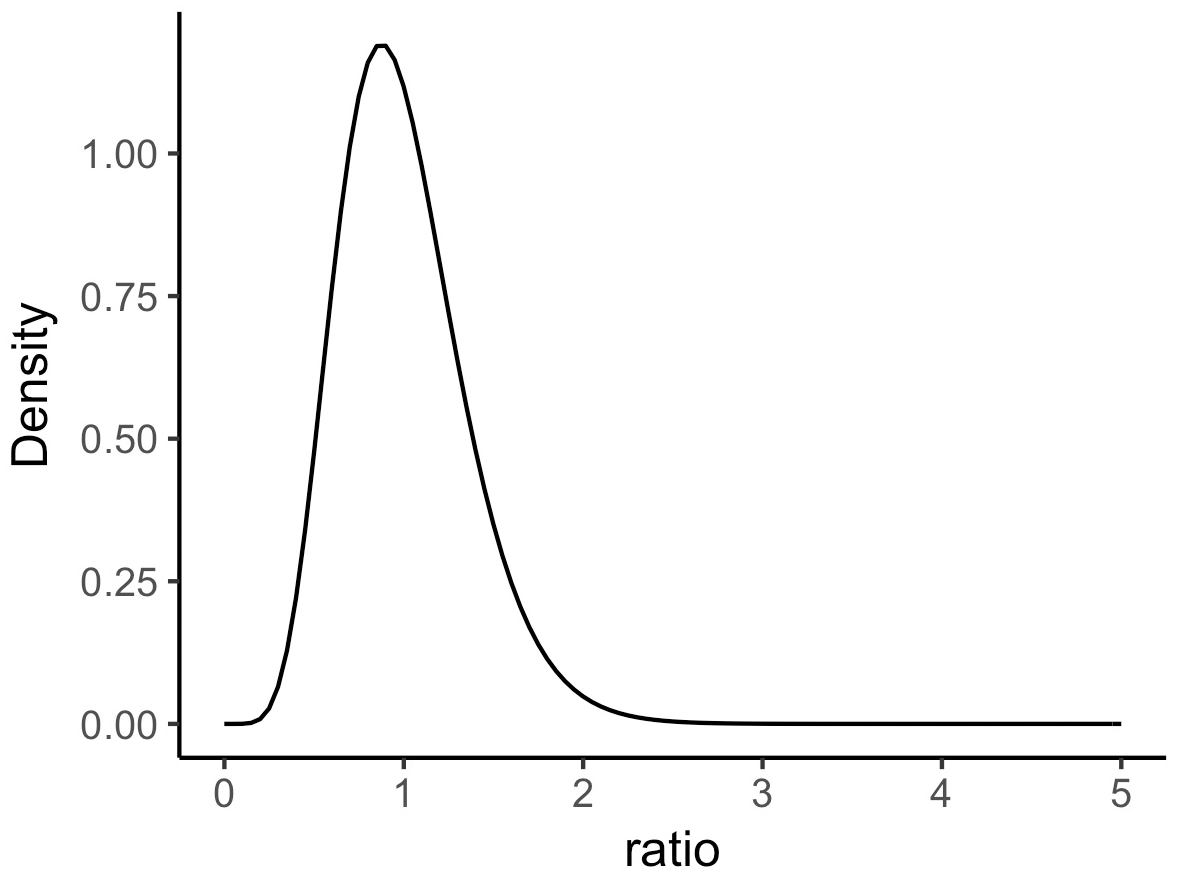
Gamma distribution used as the prior for the ratio of standard deviations. Parameters are k = 8 and *θ* = 0.125.

Following the approach for the two-sample t-test, we chose for $H_{1}$ a prior specifying the ratio between the two variances. A prior for a ratio should have a mean of 1 and has to be strictly positive. In order to meet both conditions, we chose a gamma distribution with shape k = 8 and scale *θ* = 0.125. The distribution has the form shown in Fig 4.

### Simulation results

For the Levene test, we simulated datasets with a ratio of variances varying uniformly between 1 (homogeneity) and 2. For each of two sample sizes (N = 50 and N = 200), we simulated 1000 datasets.

**Fig 5:**
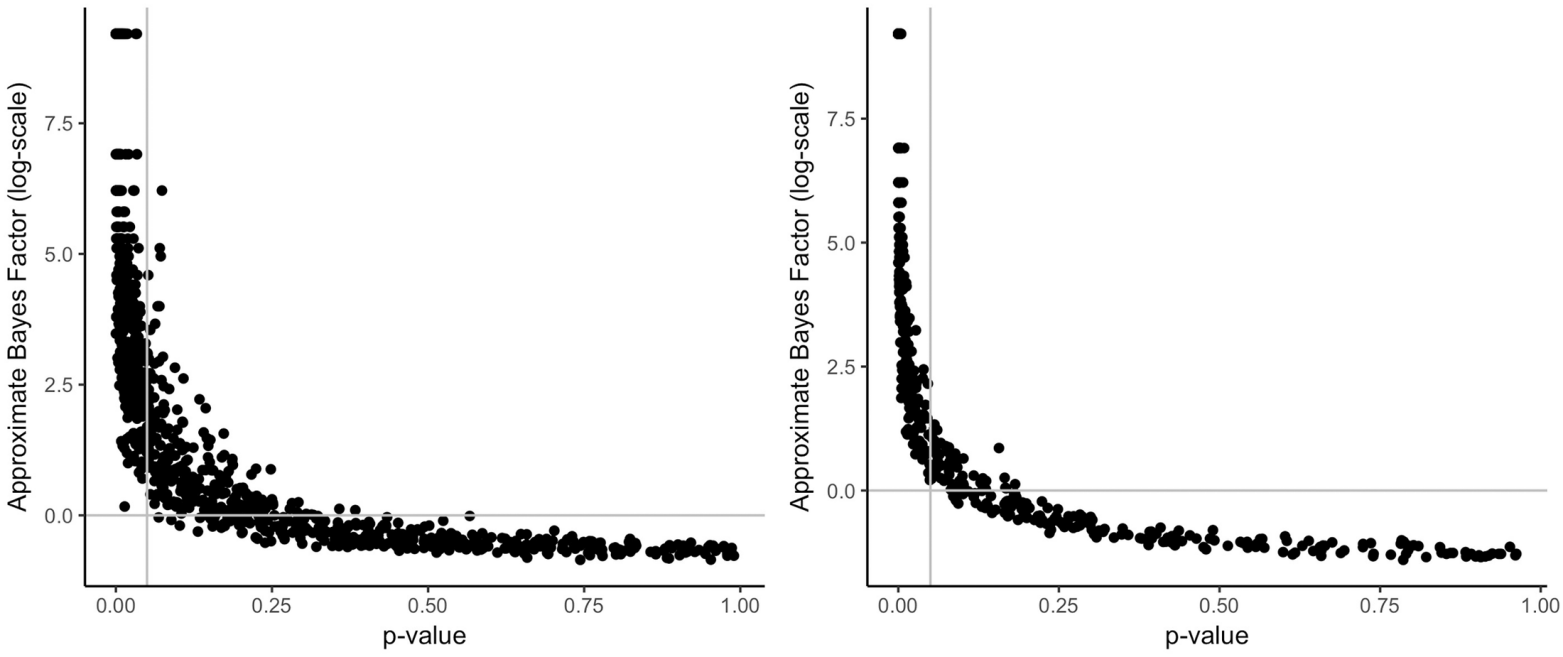
Relationship of *p*-values and approximated Bayes factors for a Levene test; left: N = 50, right: N = 200.

Fig 5 shows the relationship of *p*-values from the Levene test and approximate Bayes factors. The vertical lines in Fig 5 represent the critical value of *α* = 0.05. All *p*-values left to this line would lead to the decision of accepting $H_{1}$ (the variances are heterogeneous). The horizontal lines corresponds to a *BF*_10_ of 1. Therefore, all values above this line would indicate support for $H_{1}$, whereas all Bayes factors below this line would indicate support for the $H_{0}$ (homogeneous variances). Although both *p*-value and Bayes factor often lead to the same decision about the homogeneity of variances, according to the left plot of Fig 5, there are many datasets where decisions differ, if sample sizes are small. The area above the horizontal line and to the right of the vertical line shows datasets where there is support for heterogeneity of variances based on the Bayes factor. However, the result of the Levene test is not significant (*p* > .05).

## Application example 3: Multinomial processing trees

In this last example, we demonstrate the usefulness of ABC model comparison by computing approximate Bayes factors for multinomial processing tree models (MPT) [30]. MPT models are models for categorical data that are often used in the cognitive and social sciences. These models assume that information processing follows one of several possible paths in a so-called processing tree. For each fork in the path, probabilities are estimated. Note that recently an alternate approach to compute Bayes factors for MPT models was published [31].

**Fig 6:**
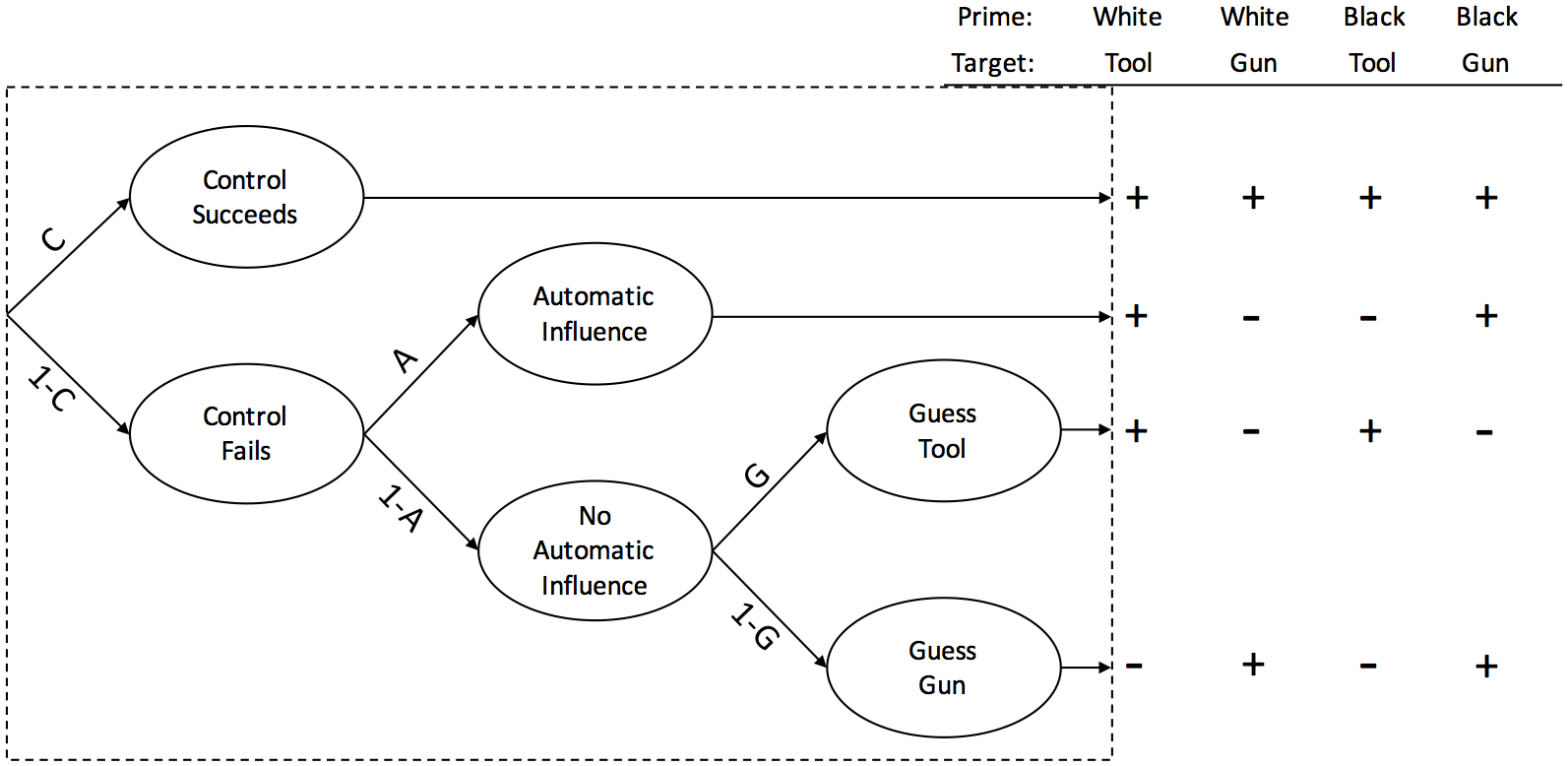
The Process Dissociation Model with guessing. Branches lead to correct (+) and incorrect (-) responses.

**Fig 7:**
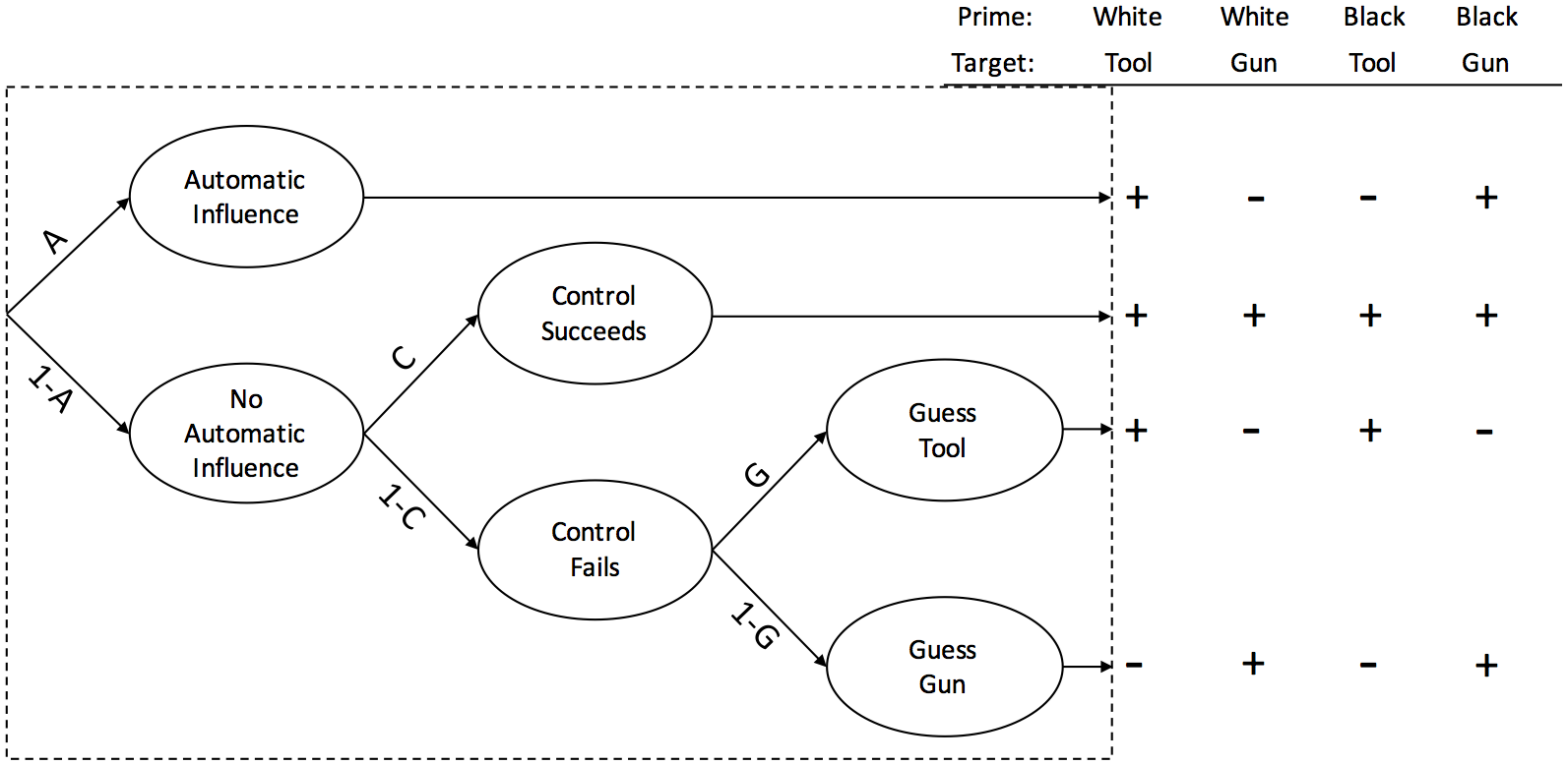
The Stroop Model with guessing. Branches lead to correct (+) and incorrect (-) responses.

For this example, we choose two prominent models that aim to determine the role of control and automaticity in the so-called weapon misidentification task [16, 17]. In this task, participants have to identify either guns or tools under high time pressure after being primed with either white or black faces. If a stereotype effect is apparent, the probability of erroneous “gun” responses is increased after black primes and vice versa. In this paradigm, it is an important question what role automatic and controlled processes play for the response selection. The Process Dissociation Model [32, 33], depicted in Fig 6, assumes that controlled processing dominates automaticity. A correct response is given whenever controlled processes prevail (probability *C*). Automatic processing, on the other hand, determines the response only if controlled processing fails *(1-C)*. The increased probability of an stereotype-consistent answer is captured in parameter *A*. A sterotype-consistent response is only correct for conditions where a gun is presented after the prime of a black face and a tool is presented after the prime of a white face. The Stroop Model [34], however, claims a reversed order of automatic and controlled processes. (see Fig 7). First, the automatic influence of a prime determines whether a stereotype-consistent response is given. With probability *(1-A)*, there is no automatic influence and controlled processing becomes relevant [16, 17].

Since MPT models can be quite complex but the data (or the simulated output of the models) only consists of counts, it is often necessary to run experiments with one or more experimental conditions in order to estimate parameters adequately. Therefore, we compare both models by implementing the design of Lambert et al. (2003) [35]. The authors of this study were interested in whether stereotypical responses are more pronounced in private settings or anticipated public settings. It was hypothesized, based on previous findings, that the anticipated public condition, in which participants were told that they had to talk to others about their results after the experiment, leads to more stereotype-consistent behavior [35]. Following Bishara and Payne (2009) [17], both models have 11 free parameters in total. Whereas the probability of controlled processing (*C*) is assumed to vary only between both conditions (private and anticipated public), the probability that the automatic response is stereotype-consistent(*A*) is allowed to vary by condition (public vs. private), prime (white vs. black) and target gender (female and male). Finally, there is one additional guessing parameter *G* that does not vary by condition.

### Prior distributions

For each of the parameters (i.e., the path probabilities), we have to set a prior distribution. Based on the parameter estimates presented in Bishara and Payne (2009) [17], we use mildly informed prior distributions. According to those results, parameter *A*, representing the probability of an stereotype-consistent answer, seems to be quite small. Therefore, a beta distribution placing more weight on small values is chosen (*α* = 2, *β* = 10). Controlled processing (parameter *C*), occurs in about half of the trials which is why we chose a symmetric beta distribution capturing this information (*α* = 3, *β* = 3). Note, that by choosing rather small values for both *α* and *β* for the prior of parameter *C*, we indicate the uncertainty regarding the parameter. In contrast, when dealing with the guessing parameter *G*, we restrict it to more narrow range around 0.5. This assumption is expressed in a beta distribution with parameters *α* = 10 and *β* = 10.

### Simulation results

In the previous simulations, we compared the Approximate Bayes factor with the default Bayes factor. For this last example, we generate 100 pseudo-observed datasets for each of the two models and compute the approximate Bayes factor. For each simulated dataset, we draw the parameters from beta distributions. The three distributions are chosen to mimic common parameter estimates (e.g. [17]).

- *A* ∼ Beta(*α* = 2, *β* = 10)
- *C* ∼ Beta(*α* = 60, *β* = 40)
- *G* ∼ Beta(*α* = 50, *β* = 50)

**Fig 8:**
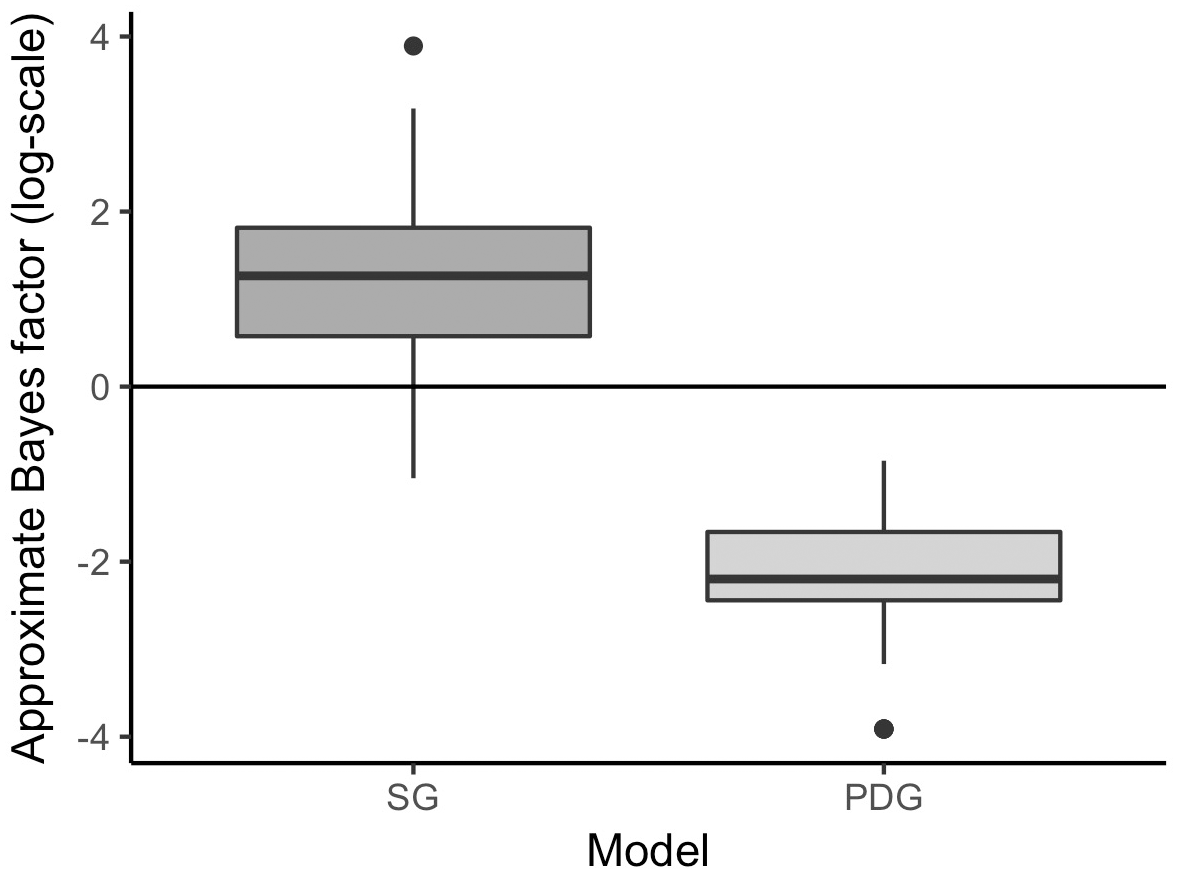
Approximate Bayes factors (log-scale) expressing support for the Stroop Model. Data are simulated from the Stroop Model (left) or the Process Dissociation Model (right).

The results shown in Fig 8. For the log-Bayes factors, positive values express the support for the Stroop Model. The left boxplot in Fig 8 shows the approximate Bayes factor when the data are simulated from the Stroop Model. As expected, most of the approximate Bayes factors are supporting the model the data have been generated from (in this case the Stroop Model). In a few cases, the approximate Bayes factor favored the PDG Model. These false decisions might be due to the fact that the threshold was set rather loose (0.05). The boxplot on the right side of Fig 8 shows the approximate Bayes factors expressing support for the Stroop Model when the data are simulated from the Process Dissociation Model. Thus, values below zero indicate support for the Process Dissociation Model. The pattern of results is similar to the one on the left-hand side. All calculated Bayes factors favor the Process Dissociation Model.

## Concluding remarks

This article introduces the reader to *ABrox*, a python package for approximate Bayesian computation with a user-friendly interface. We demonstrated the similarity of approximate and default Bayes Factor on two prominent statistical tests and demonstrated the flexibility of ABC on a comparison of multinomial processing tree models. *ABrox* defaults to the rejection algorithm for both parameter inference and model choice. However, more sophisticated algorithms are implemented. For parameter inference, a Markov chain Monte Carlo (MCMC) based algorithm by Wegmann [26] is available. Concerning model comparison, there is also the option to use random forests to calculate the posterior model probabilities [25]. Moreover, we plan to extend the set of available machine-learning algorithms for ABC by adding neural networks to the toolbox. With *ABrox*, we hope to reduce the burden for researchers to actually apply ABC methods in their labs.

## Supporting information

S1 Fig The data tab. (PNG)

S2 Fig Prior specifications in *ABrox*; example of a standard normal distribution. (PNG)

S3 Fig Function to simulate data for a model. (PNG)

S4 Fig The settings tab in *ABrox*. (PNG)
