# Supplementary figures and images for: ABrox—A user-friendly Python module for approximate Bayesian computation with a focus on model comparison
Source: PLoS One. 2018 Mar 8;13(3):e0193981. doi: 10.1371/journal.pone.0193981 (PMC5843277; doi:10.1371/journal.pone.0193981)

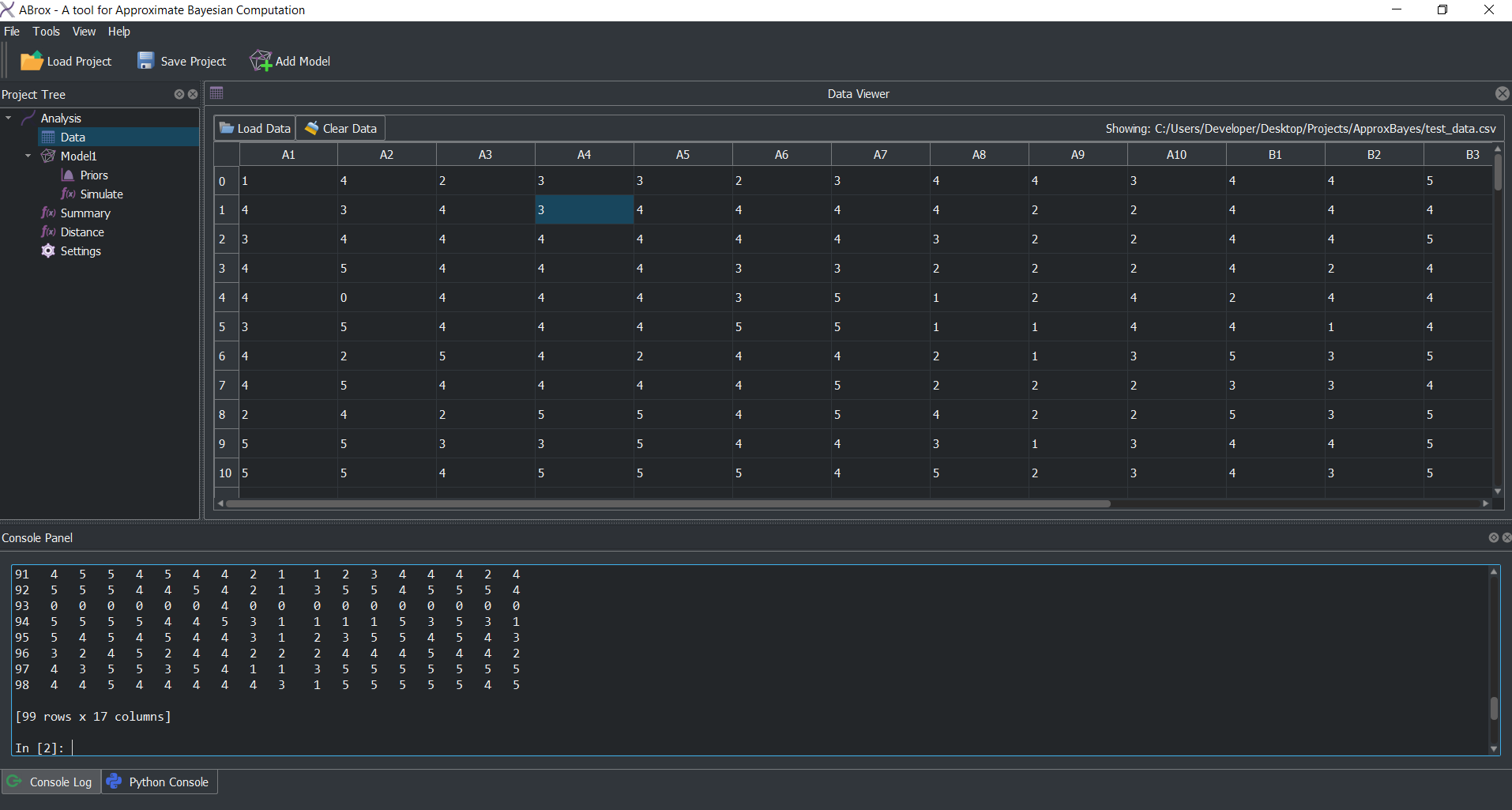

Supplement: S1 Fig — (PNG) [file pone.0193981.s001.PNG]

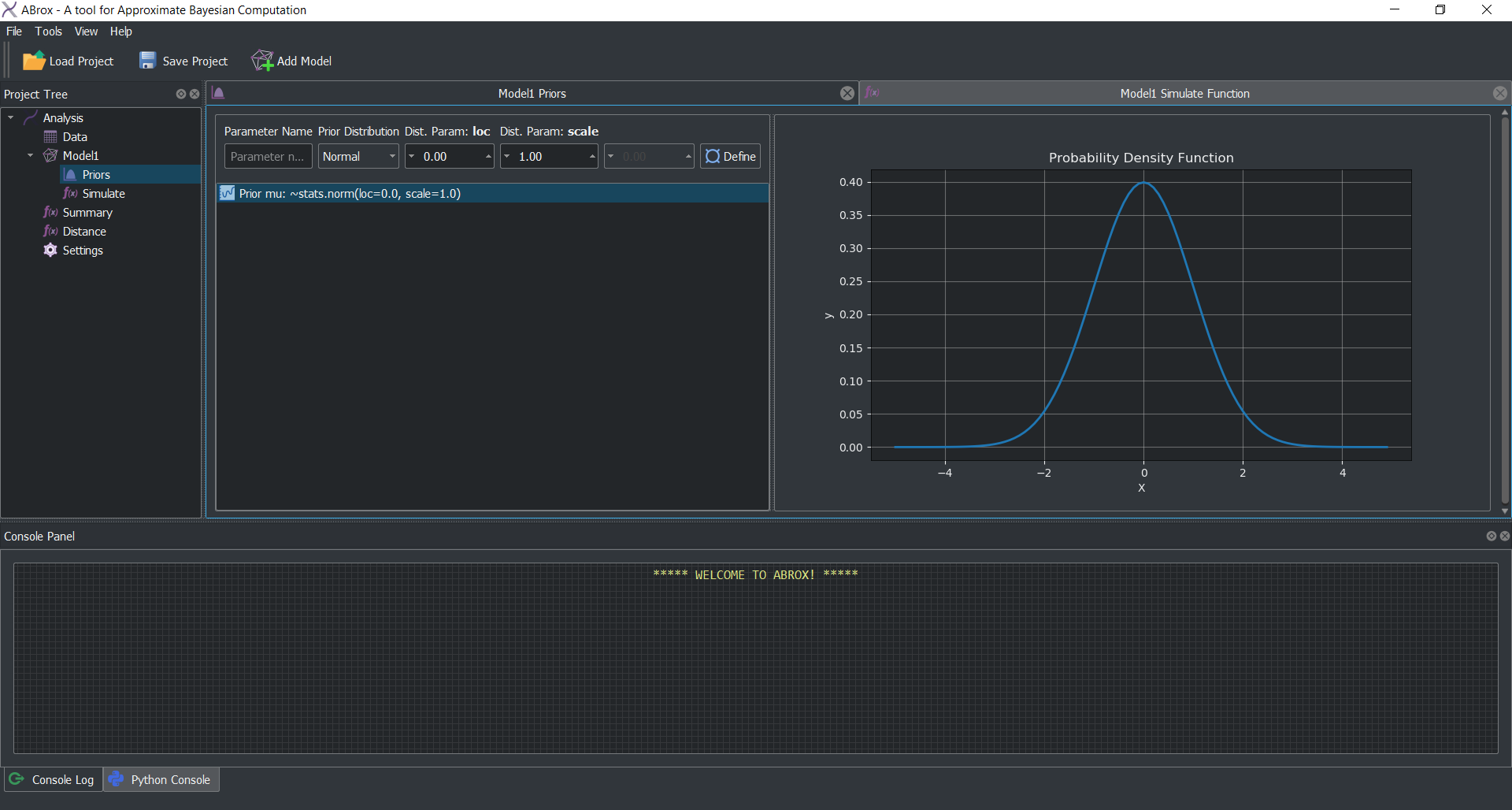

Supplement: S2 Fig — (PNG) [file pone.0193981.s002.PNG]

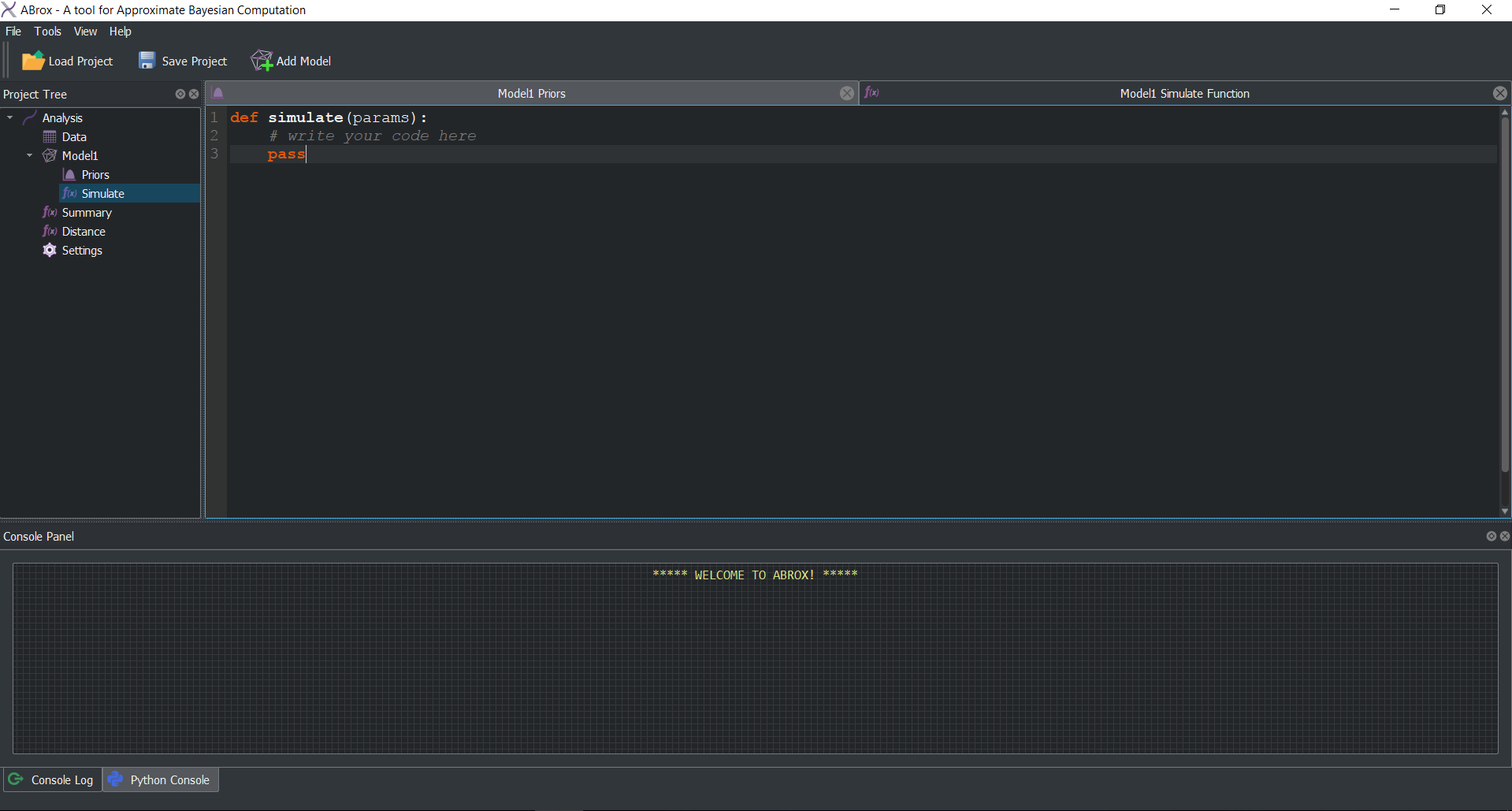

Supplement: S3 Fig — (PNG) [file pone.0193981.s003.PNG]

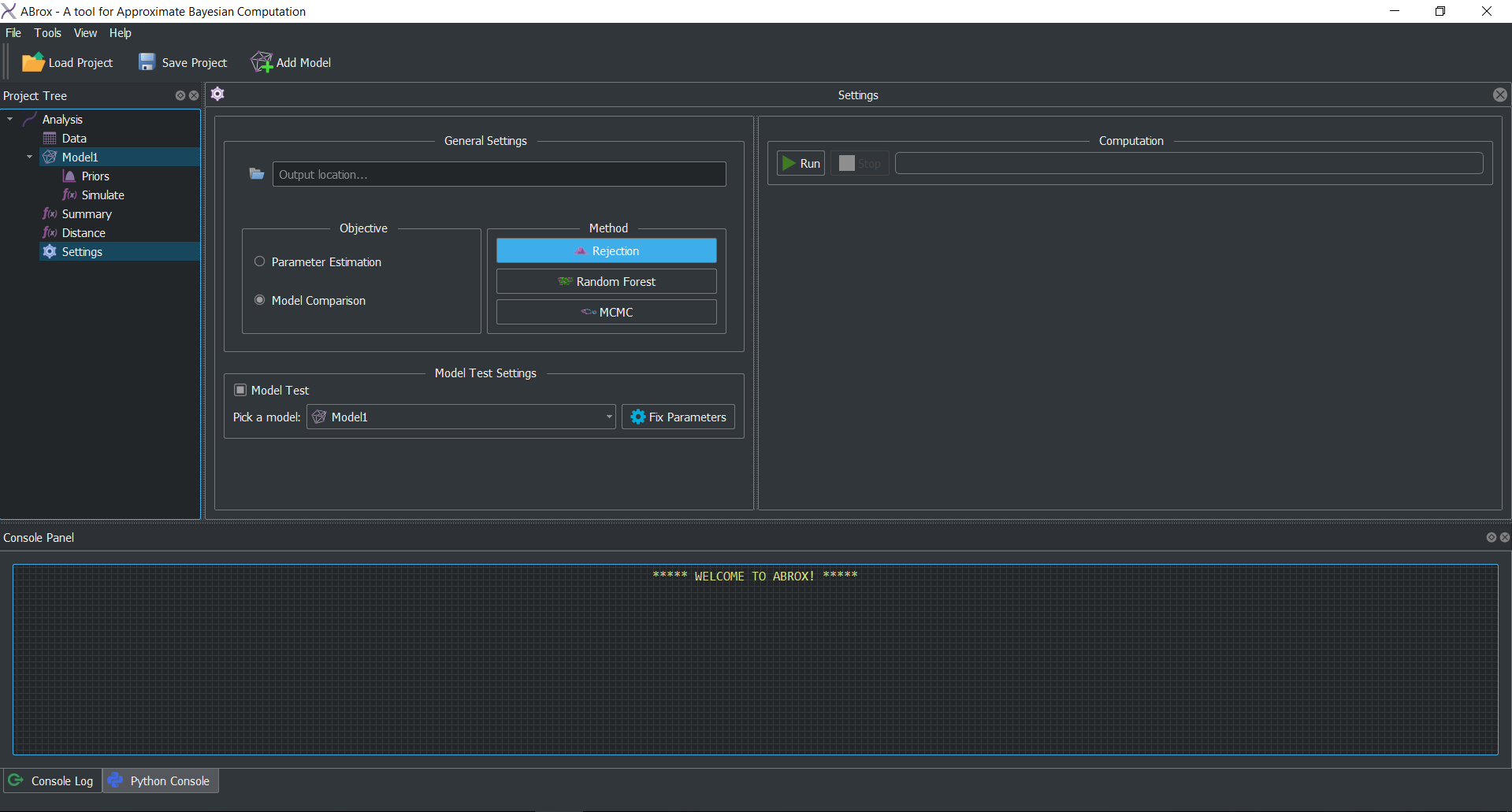

Supplement: S4 Fig — (PNG) [file pone.0193981.s004.PNG]
